# Testing the thrifty gene hypothesis: the Gly482Ser variant in *PPARGC1A *is associated with BMI in Tongans

**DOI:** 10.1186/1471-2350-12-10

**Published:** 2011-01-18

**Authors:** Sean Myles, Rod A Lea, Jun Ohashi, Geoff K Chambers, Joerg G Weiss, Emilie Hardouin, Johannes Engelken, Donia P Macartney-Coxson, David A Eccles, Izumi Naka, Ryosuke Kimura, Tsukasa Inaoka, Yasuhiro Matsumura, Mark Stoneking

**Affiliations:** 1 Institute for Genomic Diversity, Cornell University, Ithaca, NY, USA; 2 Department of Biological Statistics and Computational Biology, Cornell University, Ithaca, NY, USA; 3 Department of Biology, Acadia University, Wolfville, Canada; 4 Institute of Environmental Science and Research Ltd, Porirua, New Zealand; 5 Doctoral Program in Life System Medical Sciences, Graduate School of Comprehensive Human Sciences, University of Tsukuba, Ibaraki, Japan; 6 School of Biological Sciences, Victoria University of Wellington, New Zealand; 7 Department of Evolutionary Genetics, Max Planck Institute for Evolutionary Anthropology, Leipzig, Germany; 8 Transdisciplinary Research Organization for Subtropical and Island Studies (TRO-SIS), University of the Ryukyus, Okinawa, Japan; 9 Department of Human Ecology, Faculty of Agriculture, Saga University, Saga, Japan; 10 Faculty of Health Care, Kiryu University, Gunma, Japan; 11 Institute of Evolutionary Biology, Pompeu Fabra University, Barcelona, Spain; 12 Department of Evolutionary Genetics, Max Planck Institute for Evolutionary Biology, Plön, Germany

## Abstract

**Background:**

The thrifty gene hypothesis posits that, in populations that experienced periods of feast and famine, natural selection favoured individuals carrying thrifty alleles that promote the storage of fat and energy. Polynesians likely experienced long periods of cold stress and starvation during their settlement of the Pacific and today have high rates of obesity and type 2 diabetes (T2DM), possibly due to past positive selection for thrifty alleles. Alternatively, T2DM risk alleles may simply have drifted to high frequency in Polynesians. To identify thrifty alleles in Polynesians, we previously examined evidence of positive selection on T2DM-associated SNPs and identified a T2DM risk allele at unusually high frequency in Polynesians. We suggested that the risk allele of the Gly482Ser variant in the *PPARGC1A *gene was driven to high frequency in Polynesians by positive selection and therefore possibly represented a thrifty allele in the Pacific.

**Methods:**

Here we examine whether *PPARGC1A *is a thrifty gene in Pacific populations by testing for an association between Gly482Ser genotypes and BMI in two Pacific populations (Maori and Tongans) and by evaluating the frequency of the risk allele of the Gly482Ser variant in a sample of worldwide populations.

**Results:**

We find that the Gly482Ser variant is associated with BMI in Tongans but not in Maori. In a sample of 58 populations worldwide, we also show that the 482Ser risk allele reaches its highest frequency in the Pacific.

**Conclusion:**

The association between Gly482Ser genotypes and BMI in Tongans together with the worldwide frequency distribution of the Gly482Ser risk allele suggests that *PPARGC1A *remains a candidate thrifty gene in Pacific populations.

## Background

The prevalence of type 2 diabetes mellitus (T2DM) in Polynesians is among the highest in the world, reaching 40% among adults on some islands [1-3]. The thrifty gene hypothesis [4] offers a possible explanation for the high prevalence of T2DM in the Pacific. This hypothesis suggests that populations whose ancestral environments were characterized by periods of feast and famine experienced positive selection for alleles that promote the storage of fat and energy. Moreover, under modern conditions, populations with such thrifty metabolisms are expected to have high rates of T2DM and related traits (e.g. obesity). The ancestors of present-day Polynesians embarked on long open ocean voyages and are believed to have been exposed to cold stress and starvation during their settlement of the Pacific. Such conditions may have driven thrifty alleles to high frequency in Polynesians, thereby explaining the high prevalence of T2DM and obesity in Polynesia today [5-7].

According to this hypothesis, T2DM risk alleles at unusually high frequencies in Polynesians would represent strong candidate thrifty alleles: they may have been driven to high frequency by positive selection, thereby accounting for the high prevalence of T2DM in Polynesia. Alternatively, T2DM risk alleles may have existed as neutral alleles in the ancestors of Polynesians, and then increased in frequency in Polynesia via genetic drift, especially as a consequence of founder effects and bottleneck events. It is well-established that the ancestors of Polynesians migrated out of Taiwan through island southeast Asia and coastal and island Melanesia [8,9], and that this migration was accompanied by admixture with Melanesians and by severe reductions in genetic variation [10-13]. Such decreases in population size could have elevated the frequency of neutral T2DM risk alleles in Polynesians by chance, which then only became detrimental when Polynesians adopted a more Western diet.

Under either hypothesis, the frequency of causal T2DM risk alleles in Polynesians should be elevated, relative to their ancestral populations (East Asians and Melanesians). We therefore previously undertook an evolutionary genetics approach and genotyped 10 T2DM-associated single nucleotide polymorphisms (SNPs) in samples of Polynesian, Chinese, and New Guinean origin, without any associated phenotype information [14]. We identified the risk allele of the Gly482Ser SNP (rs8192678) in the *PPARGC1A *gene as a potential thrifty allele as it had a frequency of 0.717 in Polynesians but was absent in New Guineans. Such a striking difference in frequency was observed in less than 1% of ~90,000 SNPs genotyped in the same samples. Thus, we proposed that the high frequency of the *PPARGC1A *risk allele in Polynesians may contribute to the high prevalence of T2DM and T2DM-related phenotypes in Polynesians.

*PPARGC1A *plays a pivotal role in insulin signaling, mitochondrial regulation and adaptive thermogenesis [15-17] and is a reasonable thrifty gene candidate as it integrates metabolic pathways that support mammalian survival during prolonged starvation or hibernation [18,19]. The T2DM risk allele at *PPARGC1A *changes a glycine to serine at codon 482. The Gly482Ser SNP appears to be functional as transfection assays have demonstrated that it affects the protein's efficiency as a coactivator on the Tfam promoter [20] and 482Ser carriers were shown to have 60% less *PPARGC1A *expression in skeletal muscle than noncarriers [21]. Associations between Gly482Ser and T2DM-related phenotypes have been reported in several populations [22-28], but the absence of significant associations has also been reported [29-33]. In the present study, we further test the thrifty gene hypothesis in Pacific populations by examining the relationship between Gly482Ser genotypes and body mass index (BMI) in Maori and Tongans and by comparing the frequency of the 482Ser risk allele in Pacific populations to populations worldwide.

## Methods

Written informed consent was obtained from all participants in the present study. For the Tongans, ethics approval was obtained from the Research Ethics Committee of the Faculty of Medicine at the University of Tokyo and the Graduate School of Comprehensive Human Sciences at University of Tsukuba. For the Maori, ethics approval was obtained from the Multiregional Ethics Committee of New Zealand as part of the Rakaipaaka Health and Ancestry Study (Ethics application MEC022005) and ethical clearance was granted by the tribal organisation (Te Iwi o Rakaipaaka Inc.).

Genotype data from the Gly482Ser SNP from Cook Islanders, Western Samoans, Nuie Islanders and New Guineans (non-CEPH panel samples) was obtained from Myles et al. [14]. Atypical and related individuals from the Human Genome Diversity Panel of the Centre D'Etude du Polymorphisme Humain (CEPH-HGDP) were removed, which resulted in 952 individuals from 53 populations [34,35]. Genotyping of the Gly482Ser SNP (dbSNP ID rs8192678) in the CEPH-HGDP and the Tikopian samples was done according to the procedure described in Myles et al. [14]. The Tongan samples were genotyped with a TaqMan assay.

New Zealand DNA samples were collected from indigenous Maori individuals residing in the North Island as part of a community health survey. Individuals were unrelated by first degree, had two Maori parents by self-report, and belonged to one segment of the wider Maori population. DNA was extracted from blood using Qiagen kits and the Gly482Ser SNP was genotyped using the Sequenom platform. Evidence of association between body mass index, age, sex, population, and genotype were analysed using ANOVA and multiple regression in R.

## Results

The association analysis was performed in two Pacific populations for which BMI measures were available: Tongans and Maori. Details of the cohorts and the results of the association analysis between Gly482Ser genotypes and BMI are presented in Table 1. The mean BMI values in the Tongan (34.0 kg/m^2^) and the Maori (32.1 kg/m^2^) samples fall within the range of "Obesity class I" according to the World Health Organization [36] and are similar to BMI values observed in other Pacific populations [37-39]. We find that BMI is significantly higher in Tongans than in the Maori (two-sided t-test, *P *= 0.033). The frequency of the 482Ser risk allele is higher in Maori (0.833) than in Tongans (0.592).

**Table 1:** Summary statistics of cohorts and results of association between Gly482Ser genotypes and BMI.

							Co-dominant		Dominant		Recessive	
**Population**	**sample size**	**male**	**female**	mean age*	mean_BMI* (kg/m^**2**^**)**	**risk allele frequency**	**effect estimate (kg/m**^**2**^**)**^**#**^	***P***	**effect estimate (kg/m**^**2**^**)**^**#**^	***P***	**effect estimate (kg/m**^**2**^**)**^**#**^	***P***

Maori	110	32	78	48.1 ± 17.7	32.1 ± 7.6	0.833	0.024 ± 1.41	0.99	0.146 ± 1.60	0.93	-0.907 ± 4.52	0.84

Tongans	184	63	121	46.3 ± 14.0	34.0 ± 6.3	0.592	1.397 ± 0.66	**0.037**	2.341 ± 0.95	**0.014**	0.940 ± 1.26	0.46

All	294	95	199	47.0 ± 15.5	33.3 ± 6.9	0.686	0.351 ± 0.60	0.56	0.735 ± 0.81	0.366	-0.248 ± 1.29	0.85

* Standard deviation is shown.

^# ^Standard error is shown.

The linear regression model used to produce effect estimates included age and sex as covariates. In the analysis including all populations, "population" was also included as a covariate. Results for all three modes of inheritance (co-dominant, dominant and recessive) are shown. Statistically significant *P *values (*P *< 0.05) are shown in bold.

It is unclear from previous studies through what mode of inheritance (e.g. dominance, co-dominance or recessive) the Gly482Ser most likely acts. We therefore present results from all three possible modes of inheritance in Table 1. Age and sex were included as covariates when assessing associations within each population. We compared the results from a multiple linear regression model with age, sex and population as factors and BMI as the dependent variable to a model including only age and sex using an F test and found that the two models differed significantly (*P *= 0.0183). Thus, in assessing the relationship between the Gly482Ser genotypes and BMI using multiple regression, we performed the association within each population separately. When the analysis was performed on all of the samples together, population was included as a covariate.

**Figure 1:**
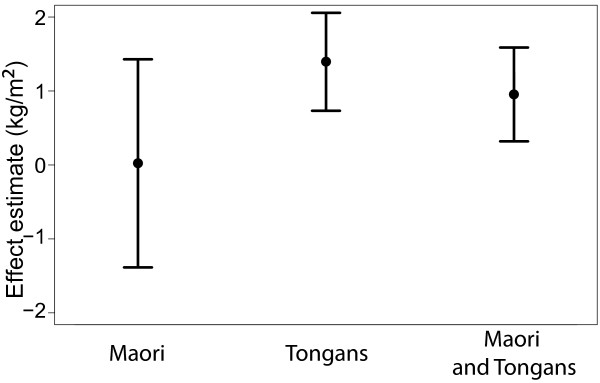
**Estimated effects of Gly482Ser SNP on BMI**. The solid circles represent the estimate and the whiskers represent the standard errors around each estimate. Estimates were obtained from a linear regression model assuming codominance of the risk allele with age and sex as covariates. In the analysis involving both populations, "population" was also included as a covariate.

Gly482Ser genotypes showed no effect on age and sex adjusted BMI in the Maori, regardless of the mode of inheritance assumed (Table 1). In the Tongans, however, the 482Ser risk allele was significantly associated with age and sex adjusted BMI under both a co-dominance model (effect estimate = 1.397 kg/m^2^, *P *= 0.037) and a dominance model (effect estimate = 2.341 kg/m^2^, *P *= 0.014), while no significant effect was observed in Tongans under a recessive model (*P *= 0.46). When the two populations were combined, Gly482Ser genotypes showed no effect on age and sex adjusted BMI under any of the three modes of inheritance (Table 1). We present the effect estimates and their standard errors for a co-dominance model in Figure 1.

**Figure 2:**
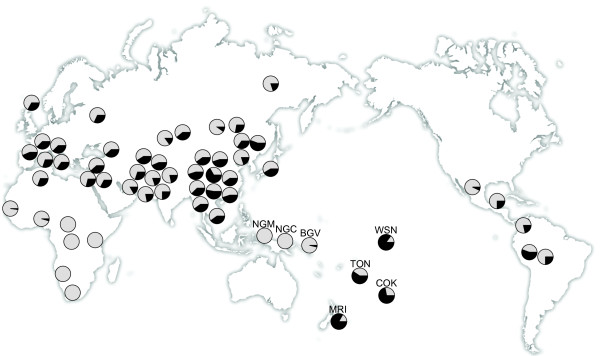
**Worldwide frequency distribution of the 482Ser risk allele**. The frequency of the 482Ser risk allele in each population is indicated in black. Frequencies are shown for the 53 populations from the CEPH Human Genome Diversity Panel and 6 additional populations. The raw genotype and allele frequency data for these populations can be found in Additional File 1, Table S1. The populations most relevant to the present study are abbreviated as follows: NGM, New Guinea samples from Myles et al. (2007); NGC, New Guinea samples from the CEPH-HGDP; BGV, Bougainville; TON, Tonga; WSN, Western Samoa and Niue; COK, Cook Islands; MRI, Maori.

The allele frequencies of the 482Ser risk allele across 58 worldwide populations, including the Tongans and Maori from the present study, are presented in Figure 2. The geographic locations, sample sizes, genotype frequencies, allele frequencies and the *P *values from Hardy-Weinberg equilibrium tests for these 58 populations are presented in Additional File 1, Table S1. The Gly482Ser SNP was in Hardy-Weinberg equilibrium in all populations tested. The frequency of the 482Ser risk allele varies widely worldwide: it is completely absent in most of Africa and in New Guinea but reaches a frequency of 0.85 in Western Samoan and Niue Islanders (Figure 2). Throughout Europe, East Asia and the Americas, the risk allele is generally found at either low or intermediate frequencies. The highest frequencies of the risk allele are observed in Polynesians.

## Discussion

Disease risk alleles with large frequency differences between populations may account for large differences in the prevalence of diseases between populations [40]. In some cases, large risk allele frequency differences between populations may be the result of local positive selection [41,42], as predicted by the thrifty gene hypothesis [4]. We previously identified the T2DM risk allele of the Gly482Ser variant in the *PPARGC1A *gene as a potential thrifty allele because of its unusually high frequency in Polynesians [14]. In the present study, we further test the thrifty gene hypothesis in Polynesians by evaluating the association between the 482Ser risk allele and a T2DM-related phenotype, BMI, in Tongans and Maori. We find that the 482Ser risk allele is associated with age and sex adjusted BMI in Tongans assuming a dominance (*P *= 0.014) or a co-dominance model (*P = *0.037), but not a recessive model of inheritance (*P *= 0.46). In the Maori, however, we found no significant associations between Gly482Ser genotypes and age and sex adjusted BMI (Table 1 and Figure 1). When considering the populations together and including population as a covariate, we also find no evidence of association (*P *= 0.56). Despite having a lower BMI, we find that the Maori have a higher *PPARGC1A *risk allele frequency than the Tongans. Although this observation does not support a causal relationship between the *PPARGC1A *risk allele and BMI, it can be explained by several confounding factors including sampling biases and environmental differences between populations.

Previous association studies between Gly482Ser and T2DM-related phenotypes have produced conflicting results, with some studies detecting significant effects [22-28] and others failing to detect effects [29-33]. These conflicting results and the differences in effect between populations observed in the present study may be the result of genetic by environment interactions or population-specific epistatic effects. It is worth noting that genome-wide association (GWA) studies have failed to detect associations between SNPs in or near *PPARGC1A *and T2DM [43-48] or BMI [49]. To date, however, these GWA studies have been restricted to populations relatively distantly related to Pacific populations. Moreover, in the present study, the lack of an association in Maori could be the result of reduced statistical power compared to Tongans due to the smaller sample size (N = 110) and the high risk allele frequency (0.833) in Maori. Our results provide some support for the involvement of the *PPARGC1A *Gly482Ser SNP in body weight or the pathophysiology of obesity related phenotypes in Pacific populations, but large-scale GWA studies in Pacific populations are desired to test this association more robustly.

If the 482Ser risk allele was driven to high frequency in Polynesians by positive selection, we expect its frequency to be relatively high in Polynesians compared to other populations. We previously showed that the 482Ser risk allele is found at unusually high frequency in a sample of Polynesians compared to a sample of highland New Guineans [14]. Here we extend the genotyping of this SNP to a worldwide sample and find that the highest frequencies of the 482Ser risk allele worldwide are indeed observed in Polynesian populations (Figure 2). One striking feature of the worldwide risk allele frequency distribution is the almost complete absence of the risk allele in Africa: we observe only three Gly482Ser heterozygotes out of 106 African individuals resulting in an allele frequency of only 0.01 in Africa (Figure 2 and Additional File 1, Table S1). Outside of Africa, the risk allele is generally found at intermediate frequencies: 0.36 in Europe; 0.33 in the Middle East; 0.27 in Central and South Asia; 0.39 in East Asia; and 0.25 in the Americas. The exception to this pattern is in Oceania where only a single heterozygote from Bougainville was observed among 53 Melanesians while the risk allele is found at a frequency of 0.69 among Polynesians (Figure 2 and Additional File 1, Table S1). The highest frequency of the risk allele worldwide is 0.85 and is found in Western Samoan and Niue Islanders. Such a striking difference in frequency between Melanesians and Polynesians is consistent with our previous observations [14] and suggests that local positive selection may be responsible for the increase in frequency of the risk allele in Polynesians. Alternatively, the non-risk allele may have gone to fixation due to selection in Melanesians. To determine whether the observed pattern is in fact due to positive selection, or whether it is the result of demographic effects such as a population bottleneck [11], will require more formal tests of selection at this locus.

It is worth noting that the search for thrifty alleles in Polynesians has not been restricted to the PPARGC1A gene. For example, it was recently shown that the Q223R (rs1137101) variant in the leptin receptor gene (LEPR) is associated with BMI and obesity in Pacific Islanders and thus represents a candidate thrifty locus [50]. However, the frequency of the 223Q risk allele in Polynesians (0.10) is relatively low: 40 of 54 worldwide populations have higher frequencies of the risk allele than Polynesians [51]. This observation suggests that the 223Q risk allele in LEPR did not rise in frequency in the Pacific due to selection and is thus unlikely to account for the unusually high prevalence of T2DM and obesity-related phenotypes in Polynesians.

The *FTO *gene was also evaluated as a candidate thrifty gene in Polynesians. Recent GWA studies conducted in populations of European origin have identified several SNPs associated with BMI in the *FTO *gene [52-54]. In Pacific populations, however, no association was detected between *FTO *risk alleles and BMI [55]. In addition, the *FTO *risk alleles are not at unusually high frequency in the Pacific [55]. These observations suggest that *FTO *is also unlikely to be a thrifty gene in Pacific populations.

## Conclusions

The thrifty gene hypothesis is an oft-cited but often untested hypothesis that provides an evolutionary explanation for contemporary patterns of disease prevalence across human populations. Our finding that Gly482Ser genotypes in the *PPARGC1A *gene are associated with BMI in a Pacific population together with the observation that the frequencies of the 482Ser risk allele are highest in Polynesians, suggest that *PPARGC1A *remains a strong candidate thrifty gene in the Pacific. Moreover, *PPARGC1A *was originally identified as a possible candidate thrifty gene in Polynesians by purely genetic methods, without any associated phenotypic data [14]; the fact that Gly482Ser is associated with BMI in Tongans supports the utility of such approaches for identifying potential candidate genes.

## Competing interests

The authors declare that they have no competing interests.

## Authors' contributions

SM and MS designed the study. RAL, GKC, DPM and DAE provided genotype data from the Maori. JO, IN, RK, TI and YM provided genotype data from the Tongans. JGW, EH and JE provided the remaining genotype data. SM performed analyses. SM and MS wrote the manuscript. All authors read and approved the final manuscript.

## Pre-publication history

The pre-publication history for this paper can be accessed here:

http://www.biomedcentral.com/1471-2350/12/10/prepub

## Supplementary Material

Additional file 1 **Table S1: Summary of the Gly482Ser genotype data used in the present study**. The geographic origin, genotype frequencies, allele frequencies and the P values from a test of Hardy-Weinberg equilibrium are presented for each of the populations genotyped for the Gly482Ser SNP in the present study. Click here for file
